# Increased songbird nest depredation due to Aleppo pine (*Pinus halepensis*) encroachment in Mediterranean shrubland

**DOI:** 10.1186/s12898-019-0270-8

**Published:** 2019-12-17

**Authors:** Asaf Ben-David, Hila Shamon, Ido Izhaki, Ronny Efronny, Roi Maor, Tamar Dayan

**Affiliations:** 1 School of Zoology, The George S. Wise Faculty of Life Sciences, Tel Aviv University, Tel Aviv, Israel; 2 The Steinhardt Museum of Natural History, Tel Aviv University, Tel Aviv, Israel; 3 Smithsonian Conservation Biology Institute, National Zoological Park, Front Royal, VA USA; 4 The Department of Evolutionary and Environmental Biology, University of Haifa, Haifa, Israel; 5 Centre for Biodiversity and Environment Research, Department of Genetics, Evolution and Environment, University College London, Gower Street, London, WC1E 6BT UK

**Keywords:** Nest predation, Acoustic monitoring, Pine encroachment, *Sylvia melanocephala*, *Garrulus glandarius*

## Abstract

**Background:**

In recent decades, a decrease of passerine densities was documented in Mediterranean shrublands. At the same time, a widespread encroachment of Aleppo pines (*Pinus halepensis*) to Mediterranean shrubland occurred. Such changes in vegetation structure may affect passerine predator assemblage and densities, and in turn impact passerine densities. Depredation during the nesting season is an important factor to influence passerine population size. Understanding the effects of changes in vegetation structure (pine encroachment) on passerine nesting success is the main objective of this study. We do so by assessing the effects of Aleppo pine encroachment on Sardinian warbler (*Sylvia melanocephala*) nest depredation in Mediterranean shrublands. We examined direct and indirect predation pressures through a gradients of pine density, using four methods: (1) placing dummy nests; (2) acoustic monitoring of mobbing events; (3) direct observations on nest predation using cameras; and (4) observation of Eurasian jay (*Garrulus glandarius*) behaviour as indirect evidence of predation risk.

**Results:**

We found that Aleppo pine encroachment to Mediterranean shrublands increased nest predation by Eurasian jays. Nest predation was highest in mixed shrubland and pines. These areas are suitable for warblers but had high occurrence rate of Eurasian jays.

**Conclusions:**

Encroaching pines directly increase activity of Eurasian jays in shrubland habitats, which reduced the nesting success of Sardinian warblers. These findings are supported by multiple methodologies, illustrating different predation pressures along a gradient of pine densities in natural shrublands. Management of Aleppo pine seedlings and removal of unwanted trees in natural shrubland might mitigate arrival and expansion of predators and decrease the predation pressure on passerine nests.

## Background

Alteration of natural habitat due to anthropogenic activities is the leading cause of biodiversity loss in terrestrial habitats [1]. Globalization has promoted alien species invasion to many parts of the world, thus threatening human livelihood and biodiversity [2]. Plants constitute for the majority of biological invasions worldwide and have tremendously impacted ecosystems [3]. Plant invasion changes habitat cover and structure, creating bottom-up cascading events with adverse effects on native species [4]. Encroachment is a form of biological invasion, whereby native species spread to habitats from which they were historically absent.

In some cases, encroachment of native species to non-native habitats are a consequence of human actions or managements. For example, native conifer species encroachment to grassland, savannas and shrublands are a well-documented phenomenon across the globe [5]. In north America it is mainly attributed to lack of fire events due to human intervention, allowing conifers to expand and establish in low vegetation habitats [6–8]. Conifer encroachment changes both biotic and abiotic conditions in soils, native vegetation composition, diversity and densities of primary and secondary consumers [9–11].

Aleppo pine (*Pinus halepensis*), is a widespread species in Mediterranean forests [12], but in the Mediterranean region of Israel, native populations inhabit relatively small and restricted areas on Mt. Carmel and the Judean mountains [13]. Aleppo pines were planted in the early twentieth century across Israel on over 100,000 ha, and account for half of the planted forests in the state. Since then, a widespread encroachment of Aleppo pines into natural habitats has been observed across Israel [14], mostly into Mediterranean shrublands [15]. Whether Aleppo pine is now an invasive- or encroaching species in this region is still debated [14, 16, 17]; we take a conservative approach and consider it encroaching, following Osem et al. [15]. Planted Aleppo pines produce seeds for approximately 30 years in the Mediterranean landscapes in Israel [15], and therefore, their impact on the floral and faunal structure is expected to continue. Hence, understanding the cascading effects of such changes is significant for understanding changes in the Mediterranean ecosystem of Israel.

Birds are considered sensitive to habitat change due to their specific adaptations to vegetation types, heights and densities [18, 19], and therefore they can be used as indicators of ecosystem intactness. Changes in vegetation structure may affect species’ ability to seek shelter or cover from threats such as predators [20]. We studied the effects of pine encroachment in natural habitats on the presence of the Eurasian jay (*Garrulus glandarius atricapillus*), a common songbird nest predator [21, 22], and the indirect effect of pine encroachment on Sardinian warbler (*Sylvia melanocephala*) nest predation. We hypothesized that avian predators such as the Eurasian jay may use pines as observation points to detect nests and that consequently pine encroachment will increase predation pressure.

The breeding season is a critical phase in the annual life cycle of birds, with important consequences to population growth and survival [23–26]. Nest predation rates usually vary between 44% and 86% depending on habitat type and species [27]; vegetation structure (i.e., micro habitat) is a significant factor that may affect nest predation [28–30].

Pine encroachment to Mediterranean shrublands may have a similar effect to that of forest edges; edge effect is an ecological change that affects the community structure in the boundaries of the habitat [31]. Forest edges can change resource availability for insectivorous birds [32, 33], as well as for nest predators; edges allow nest predators to forage in habitats other than their primary natural habitat (i.e., forest species forage in shrubland on forest edge). Increased nest predation is highly associated to edges of fragmented habitats [34], and is higher in clear edges like forest to agriculture.

There are several main predators of songbird nests in forested areas and shrublands. Small mammals (e.g. mice, rats) were found to be significant nest predators in forest habitats due to lower parental activity around the nest in such habitats [35], as predatory bird species are more associated with forest edges [36]. We hypothesized that avian predators will take advantage of spreading pines to natural shrublands in our study area.

Lahti [37] suggested that nest predation probability can be determined by specific predator behavior [37]. Members of the Corvidae family are known for their learning abilities and adaptive and opportunistic skills; therefore, these species are known to take advantage of forest edges to prey in low vegetation habitats [38, 39]. Long term monitoring through biennial bird surveys (by sound and vision along fixed transects) has been ongoing since 1985 [18]. These surveys of the study area have shown an increase in Eurasian jay density and have led to our current study. Pine encroachment to natural habitats may increase edge effects thus promoting increased presence of avian predators, such as jays in the study area. Concurrent with an increase of jay density, long term surveys show a decrease in songbird nests. Here, we investigate nest predation pressure rates over a gradient of pine densities with different vegetation composition and height. Specifically, we address the following questions: (1) is there a correlation between songbird open nest predation and (a) pine density, (b) vegetation structure (c) predator community assemblage; (2) is there a correlation between nest predation pressure and presence and activity of jays.

## Results

### Egg predation in artificial nests

**Fig. 1:**
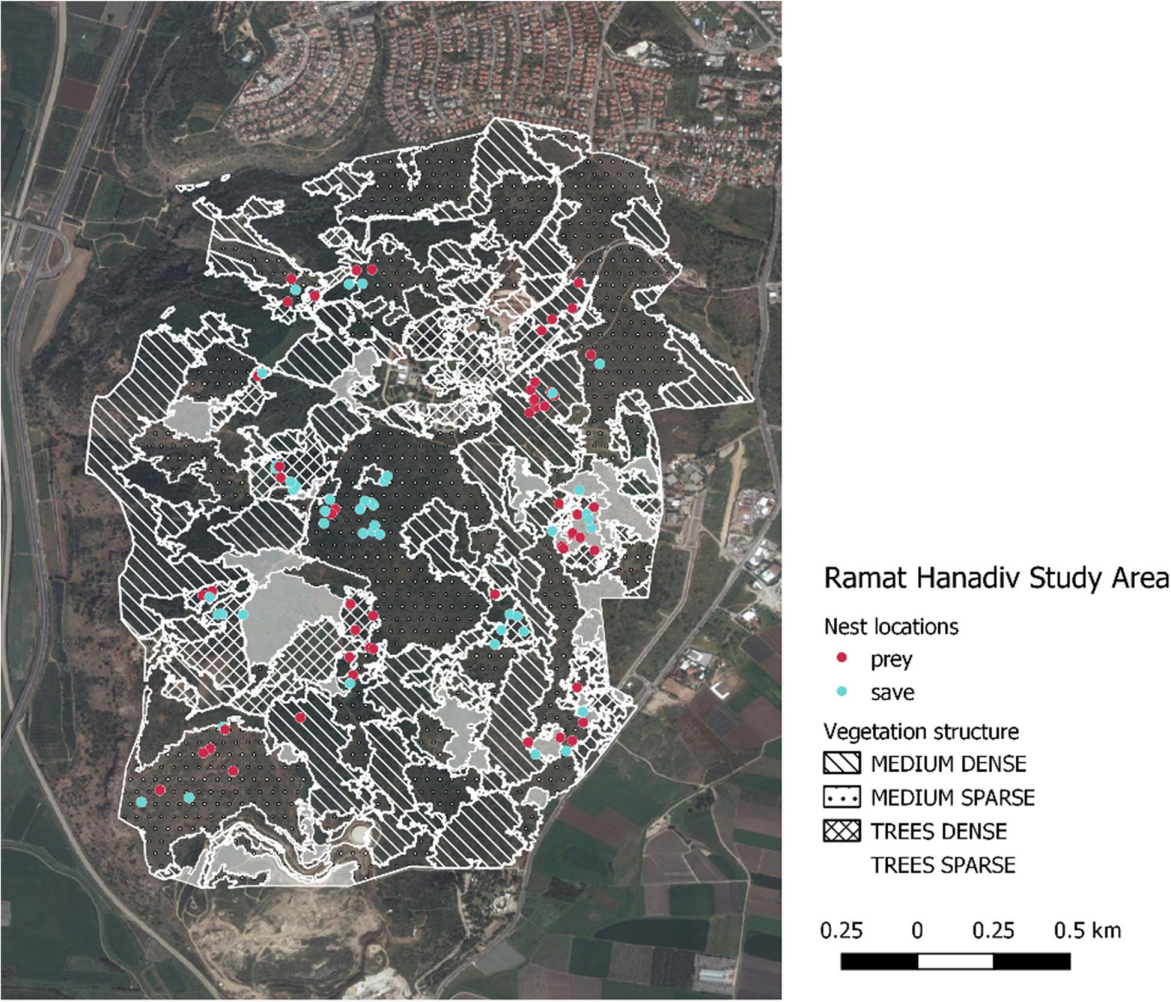
Artificial nest location at the study area, Ramat Hanadiv Nature Park, Israel. Monitoring March–May 2015–2016. Predated nests (red), saved nests (light blue). Habitat type: a shrubland, mixed scrubland and pine trees, low density pine plantation, high density pine plantation

Over half of the 123 artificial nests (quail (*Coturnix coturnix*) and plaster eggs were pooled) were predated on (65 nests, 52.8%). The probability of nest predation was not equal among the four habitat types. Greatest predation (19.5%) was recorded in the mixed shrubland and low-density pines and the lowest (7.3%) in dense pine plantations (Fig. 1).

### Nest predator identification

**Fig. 2:**
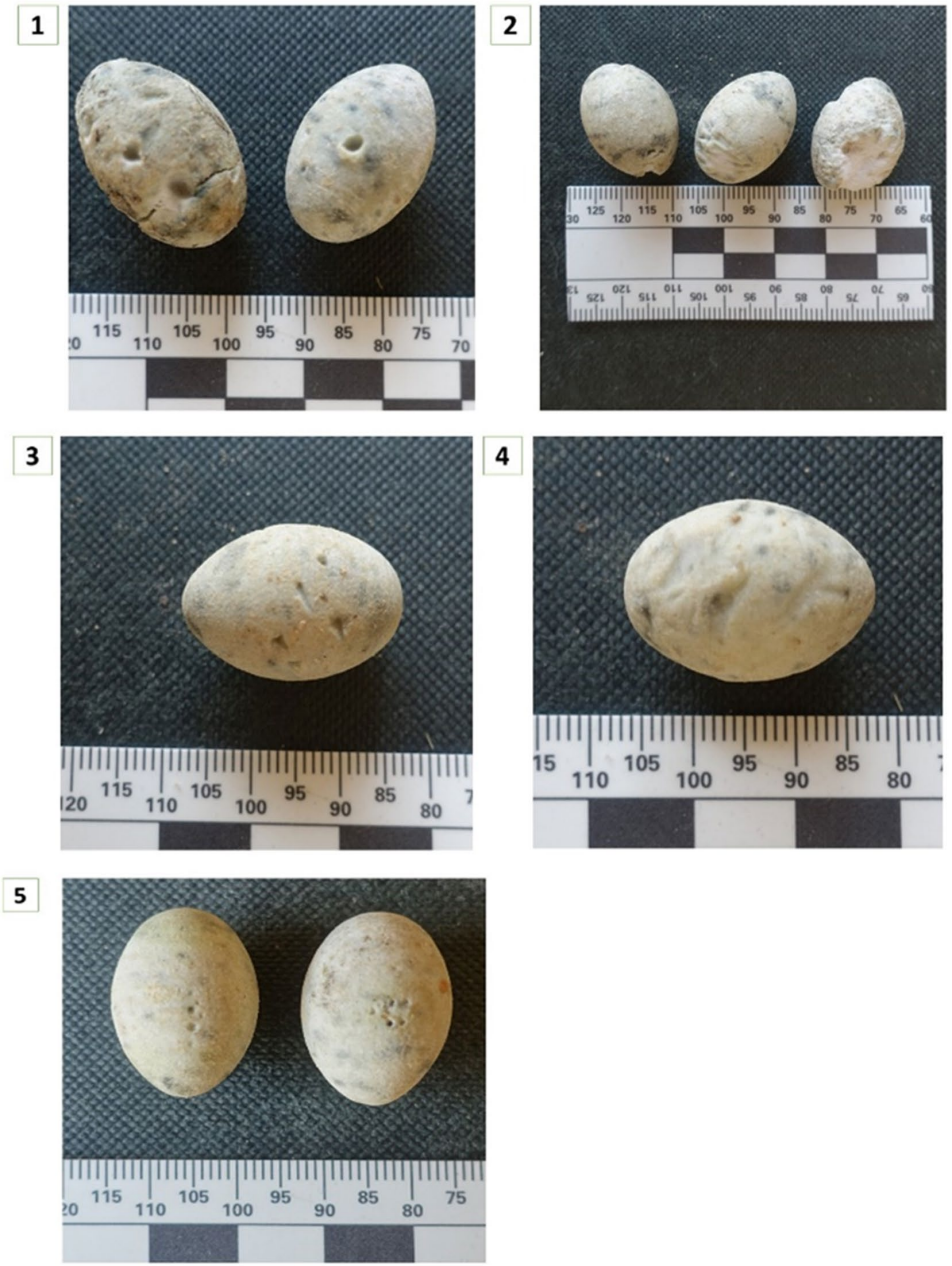
Plaster egg with teeth marks of: (1) white-breasted hedgehog (*Erinaceus concolor*)-clear mark of the canine; (2) different rodents (marks of the insectivores especially on the tip of the egg), marks of an avian predator; (3) triangle marks of bird’s nails; (4) triangle marks of a wide beak and teeth marks by a European Glass Lizard (*Pseudopus apodus*); (5) marks of row symmetric teeth similar in size

Based on the 48 plaster eggs that we placed in artificial nests, we were able to identify four predator taxa according to marks left on 18 plaster eggs, (Fig. 2). The main predator group was medium size mammals (n = 8), followed by rodents (n = 7), birds (n = 2) and reptiles (n = 1) (*Pseudopus apodus*, Eurasian Glass Lizard). Some nests that were located close to the cattle corral disappeared completely with the shrubs around the nest site (n = 3) (Fig. 2).

Our camera traps were set near artificial nests with quail eggs to learn about predator behavior: which predator species leave remains, which obtain all eggs, and which remove the entire nest. We found that jays were associated with 84% of documented predation events and 100% of those events ended up with eggs disappearing from artificial nests. A quarter of the nests (n = 12) with quail eggs were emptied from all their eggs with no visible remains. Camera trap images showed that jays carried away the eggs, small mammals bit the egg and left remains in the nest and meso-carnivores removes entire nest. Therefore, artificial plaster egg disappearance was associated with avian predator category.

**Fig. 3:**
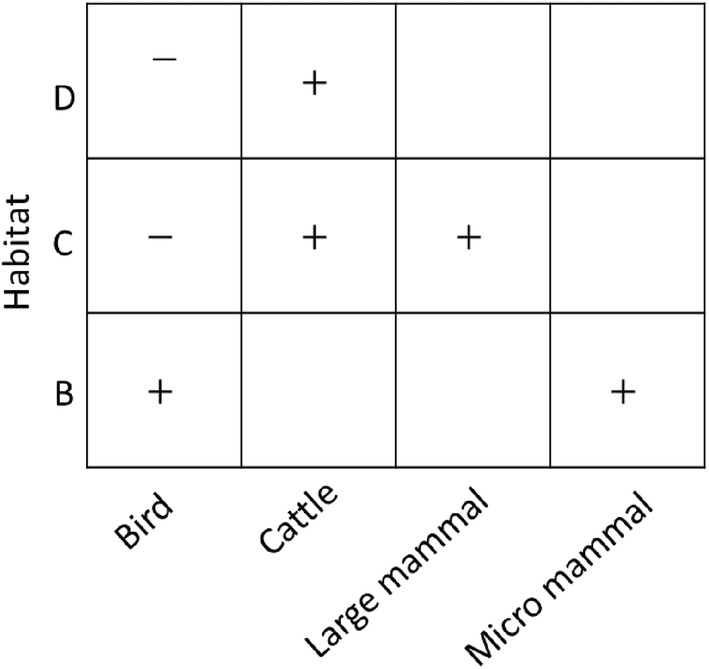
Individual predator type response to habitat type tested using a multivariate abundance model based on a generalized linear model (GLM) from data obtained by artificial plaster egg experiment. Presented are coefficient response with significance (p < 0.05) in comparison to intercept − habitat (A). Habitat type: (A − intercept) natural shrubland; (B) mixed shrubland and encroaching pines; (C) low density Aleppo pine plantation; (D) high density Aleppo pine plantation. Positive correlation (+), negative correlation (−)

Predator assemblage was significantly different between natural shrubland (A) and the three other habitat types (Mixed shrubland and encroaching pines—Wald value: 1.465, *p *<0.01; Low density Aleppo pine plantation—Wald value: 2.122, *p *<0.01; High density Aleppo pine plantation—Wald value: 1.733, *p *<0.01, Fig. 3). Avian predation was significantly higher in the natural shrubland mixed with pine than in natural shrubland, and avian predation in pine plantations was significantly lower than natural shrubland (intercept). Predation due to micro-mammals (i.e. rodents) occurred significantly more at mixed shrubland and mixed habitat and was not significantly different at the pine plantation habitat. Large mammal predation was significantly higher at low-density plantations and significantly lower at mixed shrubland and encroaching pines. Nest trampling due to cattle occurred significantly more at pine plantations than natural shrubland (intercept) (Fig. 3).

### Nest survival probability

**Fig. 4:**
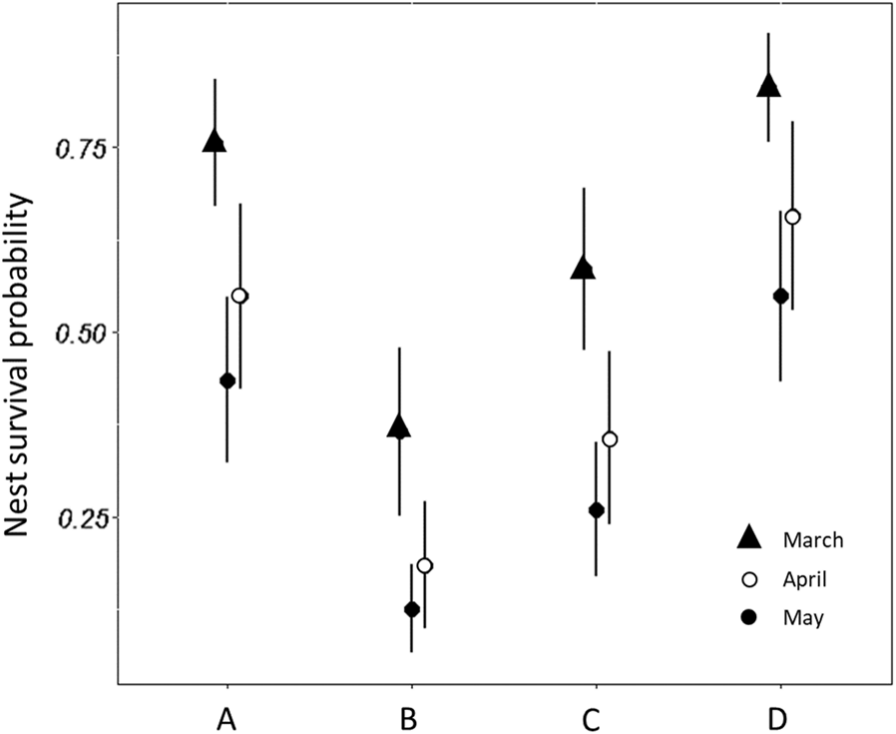
Predicted estimation of the nest survival probability in relation to habitat type and time of nesting season [month: March (triangle), April (hollow circle), May (full circle)]. Whiskers indicate lower and upper confidence intervals. Habitat types: (A) natural shrubland; (B) mixed shrubland and encroaching pines; (C) low density Aleppo pine plantation; (D) high density Aleppo pine plantation. Data collected at Ramat Hanadiv Nature Park during March–May 2015–2016

We estimated nest survival in relation to habitat type and the time within the breeding season (March, April and May). We used natural shrubland as an intercept. The GLM binominal distribution model predicted that nest survival probability was significantly lower in the mixed shrubland and encroaching pine during the whole breeding season in comparison with the other three habitat types (coeff: − 1.6814 (SE 0.58), *p *=0.016). The probability of survival was also low in the low-density pine plantations; however, this difference was not significant in comparison to the natural shrubland (coeff: − 0.7845 (SE 0.53), *p *=0.14) (Fig. 4). The probability of survival was relatively high but not significantly different between the two control habitats, natural shrubland and high-density pine plantation, respectively) (coeff: − 0.4560 (SE 0.5), *p *=0.42). Time within the nesting season (measured by month) had a significant impact on nest survival where nest was more likely to survive in the beginning of the nesting season (March) than later in the season (April and May). Lowest survival predictions (compared to March (intercept)) were estimated in April (coeff: − 1.3901 (SE 0.47), *p *=0.003), followed by May (coeff: − 0.9354 (SE 0.52), *p *=0.072) (Fig. 4).

### Indirect evidence of predation pressure

**Fig. 5:**
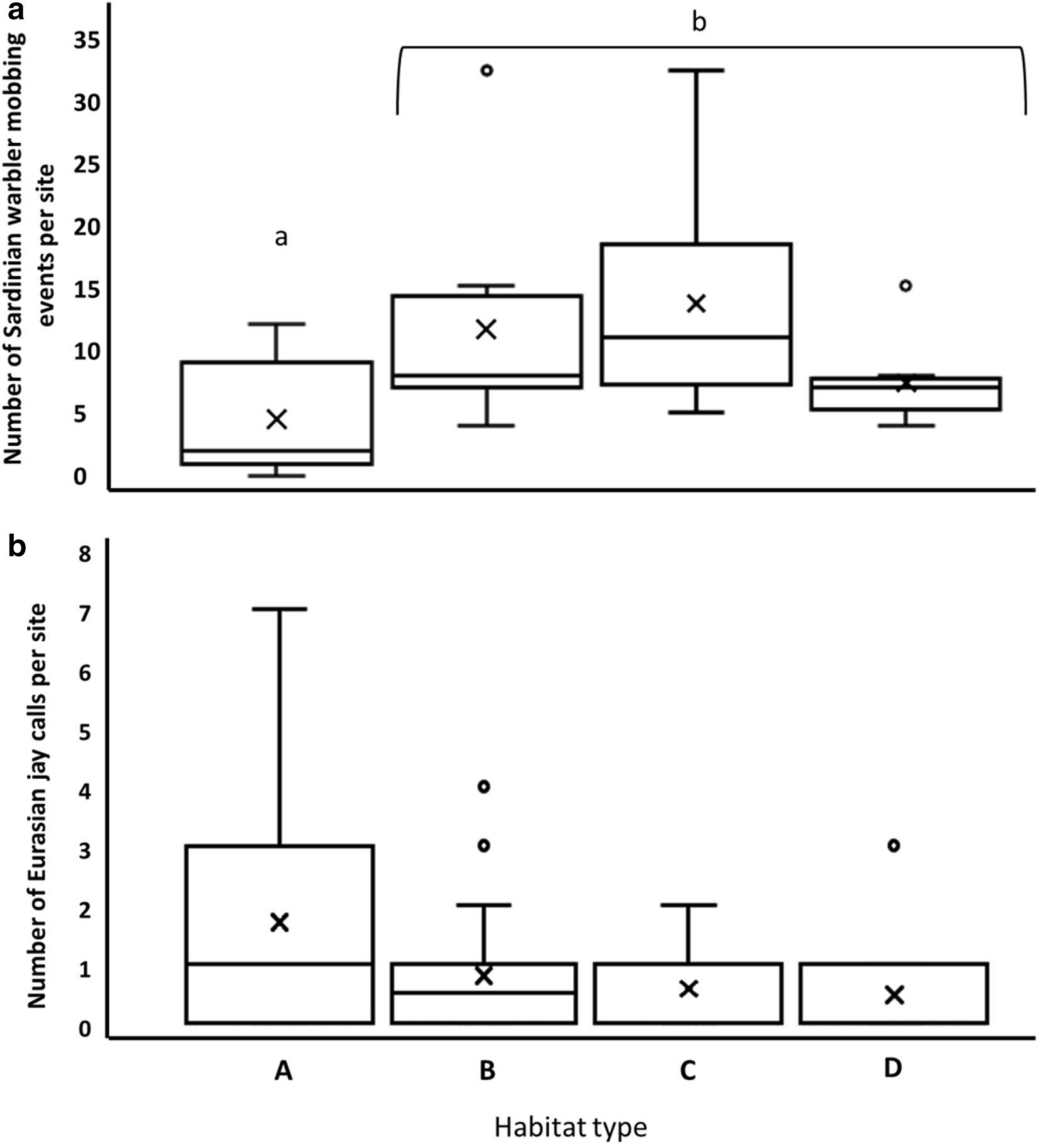
Sardinian warbler mobbing events (**a**) and Eurasian jay calls (**b**) in the four habitat types: natural shrubland; mixed shrubland and encroaching pines; low density Aleppo pine plantation; high density Aleppo pine plantation. The distribution of Sardinian warbler mobbing events (mean (×), median (solid line), post hoc difference (letters A, B), whiskers represent upper 0.95 and lower 0.05, box upper 0.75 and lower 0.25. Points represent outliers. Based on 220 h of recordings from 06:00 to 10:00 AM (n = 88). Habitat types: (A) natural shrubland; (B) mixed shrubland and encroaching pines; (C) low density Aleppo pine plantation; (D) high density Aleppo pine plantation. Data collected at Ramat Hanadiv Nature Park during March–May 2015–2016

Sardinian warbler mobbing calls represent the extent of “threat” in a given habitat and Eurasian jay calls represent predator presence in a given habitat. We analyzed 220 h of recordings from 16 different plots. We found that the number of Sardinian warbler mobbing calls was significantly different among the four habitat types (Kruskal–Wallis, *χ*^2^=15.564, *df *= 3, *p *=0.0014). Post-hoc with Wilcoxon tests and Bonferroni correction showed significantly lower number of mobbing calls in the natural shrubland in comparison to the other three habitats. The highest number of mobbing calls was detected in the mixed shrubland and pine plantation habitat (Fig. 5).

Although the number of jay calls, identified from the acoustic monitoring, was not significantly different between the four habitat types (*χ*^2^ = 6.888, df = 3, p = 0.076), the results showed higher numbers in the mixed shrubland and in the low-density pine plantation and lowest number of calls in natural shrubland.

### Direct observations

**Fig. 6:**
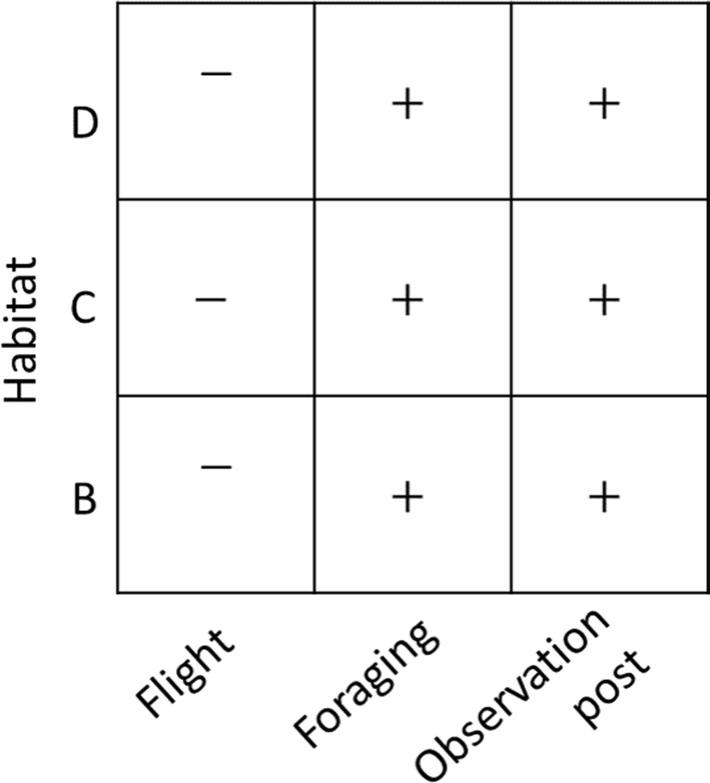
Activity type correlation to habitat type. Activity: (1) foraging; (2) flying; (3) siting on observation points (trees). Presented are coefficients with significance (p < 0.05) in comparison to intercept − habitat (A). Habitat type: (A − intercept) natural shrubland; (B) mixed shrubland and encroaching pines; (C) low density Aleppo pine plantation; (D) high density Aleppo pine plantation. Positive correlation (+), negative correlation (−)

Direct observations on fixed trails were carried out 20 times throughout the study. We recorded 67 jay occurrences in the study area and characterized them to three types of activity: (1) foraging; (2) flying; (3) observation points. We used a multi-dimensional analysis based on a GLM framework to distinguish between activity occurrences between the four habitat types. We used natural shrubland as an intercept in the model, thus the three other habitats were compared to this habitat and were found to be significantly different in terms activity type assemblage (Mixed shrubland and encroaching pines; Wald value: 2.956, p value: < 0.005); (Low density Aleppo pine plantation; Wald value: 1.717, p value: < 0.005); (High density Aleppo pine plantation; Wald value: 2.844 p value: < 0.005). We assessed goodness of fit using Likelihood Ration Test and Akaike information criterion (AIC) score, model that included habitat performed better (Jay Behaviour ~ Habitat; AIC = 69.6; Null (Intercept only): AIC=70.83; LRT=16.773, *p*(*χ*^2^)=0.05). Significantly increased foraging behavior and decreased flying behavior were observed at all three habitats in comparison to natural shrubland and was positively correlated to mixed shrubland and pines (Fig. 6). Jays were also observed significantly more on observation points in these three habitats in comparison to the natural shrubland (Fig. 6). These results indicate that jays flew over more frequently in natural shrubland without stopping to forage in this habitat.

## Discussion

We found that Aleppo pine encroachment to natural shrublands increased nest predation events by Eurasian jays due to changes in habitat structure. Our results highlight how encroaching pines can directly increase the activity of avian predators in shrubland habitats and reduce the nesting success of shrubland songbirds. These findings were based on multiple methodologies that illustrated different predation pressures among the four habitat types, including artificial nest predation, acoustic monitoring of warbler mobbing events and jay calls, and jay activity patterns in relation to a gradient of pine densities in natural shrublands.

A 30-year long term songbird nesting survey (1985–2015) carried out in this study area has demonstrated a decline in warbler and some other passerine densities, and an increase of jay densities [18]. Results of our study offer a mechanistic explanation for this decline. We expect that increased nest depredation due to forest encroachment affects other shrubland passerines that have a similar breeding strategy to that of the Sardinian warbler. Further investigation is needed to assess this hypothesis. Changes in species densities and species turnover along vegetation gradients have been linked to predation pressure [30]. In habitats that undergo rapid changes, like in cases of biological invasions [40], such changes may therefore be accelerated. We found that decrease of songbird species in areas of tree encroachment is not a direct response to vegetation change but to increased predation. The lack of parental activity around artificial nests may lower the probability of detection by predators [41], so the results we present here may underestimate the predation risk to active nests.

Birds from the Corvid family detect nests more efficiently by using elevated points such as electricity poles to observe nesting areas [42]. In the current study, it is encroaching trees that serve jays for the same purpose. Eurasian jays do not forage far from forest patches [19, 43] and our results concur; we found a high presence of jays in pine-encroached shrublands and in pine plantations, and a lower occurrence in undisturbed shrublands. Previous studies found that nest predation in open landscapes and shrublands decreased with increasing distance from forest edges [44]; our results provide a mechanistic explanation to such observations.

Conifer encroachment into shrublands can facilitate prey bird movement via “stepping stones” that the pines create [45]. Also, tree vantage points within the landscape reveal the passerine activity around the nest for jays and in mixed shrubland and pine patches. Schmidt [46] suggested in a theoretical paper to look at nest predation from an optimal foraging theory perspective. It predicts that nest predators would forage where they have the best energetic profit to their foraging efforts [46]. We suggest that Eurasian jays may profit by foraging more efficiently using vantage points on pine trees.

## Conclusions

In the last 150 years, the distribution of woody vegetation in open habitats, such as grasslands and savannas, has increased worldwide, jeopardizing biodiversity [47]. Our study demonstrates that the encroachment of Aleppo pines into shrubland habitats may promote Eurasian jay populations and those of other avian predators, and consequently negatively impact populations of open-nest passerines. Thus, our results illustrate a causal mechanism of changes in bird community, which is generalizable to other parts of the world that suffer from tree encroachment. In areas of encroachment of encroaching pines, such as the Mediterranean region, measures should be taken to control this phenomenon. A first measures would be to cease planting conifer forests for recreational use. Second, management of seedlings at existing plantation edges. Third, removal of mature encroaching trees from natural shrub habitats. We encourage land and wildlife managers to consider our conclusions for decisions in conservation management schemes of passerine populations.

## Methods

### Study area

The study was carried out in Ramat Hanadiv Park in the southern Mt. Carmel, Israel (32° 30′ N, 34° 57′ E), an area of 450 ha, surrounded by suburbs and agricultural fields. A cultivated memorial garden is located in the park center. The area is a plateau with a mean elevation of 120 m above sea level. The climate is typical Mediterranean with an average maximal temperature of 25.6 °C and minimal temperature of 16.4 °C, the average wind speed archives 9.78 m/s, rainfall averages 493 mm annually, and occurs mainly from November to March (Meteorological weather station data. http://www.meteo-tech.co.il/hanadiv/hanadiv_periodical.asp#; Accessed 12 Nov 2019). The main vegetation structure is an open shrubland with broad-leaved phillyrea (*Phillyrea latifolia*) and mastic tree (*Pistacia lentiscus*) and planted groves of Aleppo pine (*Pinus halepensis*), Turkish pine (*Pinus brutia*), and stone pine (*Pinus pinea*) [48].

### Study design

**Fig. 7:**
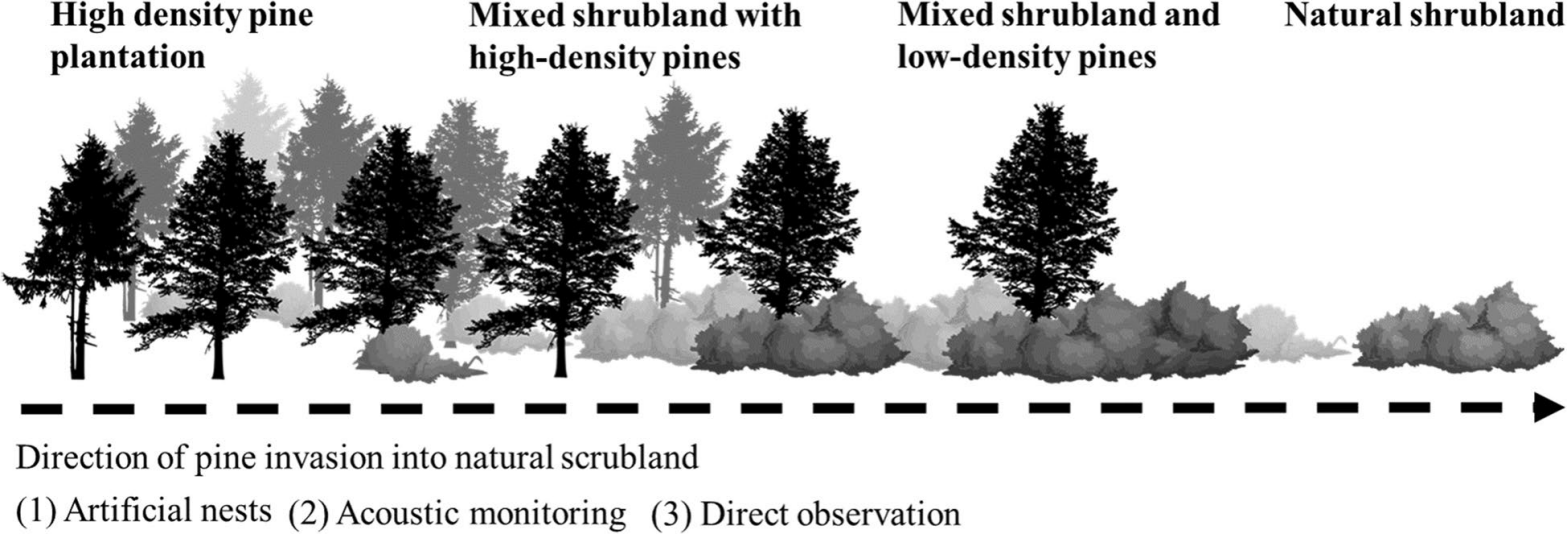
Conceptual figure of study design. Four habitat types following a gradient of pine densities: (A) natural shrubland; (B) mixed shrubland and low-density pines; (C) mixed shrubland with high-density pines; (D) high density pine plantation. In each habitat, be used three methods to monitor predation pressure and warbler and jay occurrence (artificial nests, acoustic monitoring, and direct observations)

We chose four different habitat types that differ in pine densities and height as well as in vegetation cover and composition (Table 1, Fig. 7): (1) Shrubland: an open shrub with mainly broad-leaved *P. latifolia* and *P. lentiscus*, without trees that taller than 3 m. Annual and perennial herbaceous plants occur between the shrubs; (2) Mixed shrubland with pines: an open shrub of *P. latifolia* and *P. lentiscus* with Aleppo pine trees taller than 3 m (10–15 trees per hectare). Annual and perennial herbaceous plants occur between the shrubs and trees; (3) Low density pine plantation: a mixture of pine and mastic shrubs. Annual and perennial herbaceous vegetation occurs between the shrubs. Density of 30–40 trees per hectare; (4) High density pine plantations are characterized by densities of 70–80 trees per hectare. Some Cypress trees (Cupressaceae) and few Mastic tree. We consider trees that are taller than 3 m based on Light Detection and Ranging (LiDAR) data products provided by Ramat Hanadiv. We used habitat types 1 and 4 as controls: the shrubland is a typical warbler habitat and the high-density pine plantation is a typical habitat of the Eurasian jay. Jays are highly intelligent birds that use their learning abilities [49, 50] to specialize in nest robbing, particularly in forest edges (i.e., mixed shrubland and pines). In each habitat we quantified predation pressure using three methods (detailed below): (1) artificial warbler nests to monitor nest survival and predator community; (2) monitored warbler mobbing events and jay calls using acoustic recordings to assess predation pressure and jay presence; and (3) direct observations on jay activity; (4) validation of predator events using camera traps (Fig. 7).

**Table 1:** The properties of the habitat types in Ramat Hanadiv Nature Park (following Bar Massada et al. [57])

Habitat type	% of the park area	Typical plants	Structure
Natural shrubland	35	Broad-leaved Phillyrea, Mastic tree	0.5–2 m tall, 25–50% cover
Mixed shrubland with pines	31	Broad-leaved Phillyrea and Mastic tree and few pine trees	0.5–5 < m tall, > 75% cover
Open pine plantation	4	Pine trees with Mastic tree	Height > 5 m, < 75% cover
Dense pine plantation	7.4	Pine and Cyprus trees	Height > 5 m, > 75% cover

#### Artificial nests

We randomly chose 16 study plots (4 for each habitat) of 0.2 km^2^. Four dummy nests were randomly stationed in each plot, 30–100 m apart, in two breeding seasons (2015 and 2016). The nests were built from local weeds, flax and Raffia palm fibers in diameter of 10–15 cm in a coiling basket wreathing method [51]. The size and the material of the nests were made after examining Sardinian warbler nests from the Steinhardt Museum of Natural History (Tel Aviv University, Tel Aviv, Israel) nest collection. We placed two types of eggs in these artificial nests: (1) Quail (*Coturnix coturnix*) eggs that were boiled in order to postpone their rotting; (2) 3D printed plaster eggs similar to the size and decorated texture of Sardinian warbler eggs (model Projet^®^ CJP 660Pro by 3D Systems) [3]. The artificial eggs were covered with white bee wax and mixed with droppings from a chicken (*Gallus gallus domesticus*) to mask the smell of wax. The wax was used to collect information on nest predators according to the marking left on eggs (e.g., teeth and beak marks). Each nest was placed in the thickest shrubs in each plot and was reinforced to bush branches with a zip tie at a height of 50–120 cm from the ground, similar to the natural nesting sites of Sardinian warblers [52].

Three boiled quail eggs were placed in 80 nests (16 nests in each cycle) in five different cycles (18th April to 3rd May 2015, 15th May–5th June 2015, 1st to 22nd March 2016, 10th to 30th April 2016, 6th to 27th June 2016). Each nest was set in the field for three consecutive weeks. Three plaster eggs were placed in each nest in the three experiment cycles during 2016 breeding season (1st to 22nd March 2016, 10th to 30th April 2016, 6th to 27th June 2016). Five nests fell off the shrub and were lost, leaving a total of 43 nests in these experiments. Each nest was set in the field for three consecutive weeks. Predation marks on the eggs were identified to species level by measuring incisors of the common rodent species and canines of common carnivores. For reptiles, we compared bite marks with preserved specimens in the Steinhardt Museum of Natural History skull collection. Avian marks were identified by beak and claw marks as described on acorns in birds tracks and sings field guide [53].

#### The association between predator assemblage and habitat type

We used a multivariate abundance model based on a generalized linear model (GLM) [54] to correlate between the predator assemblage and the habitat type. This high dimensional GLM analysis has been proven to have better power than common distance-based methods [55], and enables to make hypotheses about community-environment associations (both community-level and taxon-specific inferences), and estimates the direction and influence of explanatory variables have on single species and a whole communities, Model selection was conducted using a stepwise procedure and nested models were assessed by Likelihood Ratio Test and AIC score. The analysis for all taxon levels was done using ‘mvabund’ package [55] available on R [56].

#### Nest survival probability

We modeled the probability of nest survival with a binominal generalized linear model (GLM) using the “stats” package in *R* v3.2.3 [56]. First, we compared predation rates upon the plaster and the quail eggs and found no significant difference using Chi square with Monte Carlo simulation (due to the small sample) (*n *=123, *df *=3, *p *= 0.86). Therefore, the experiments with the two types of eggs were pooled prior to the GLM analysis. Model explanatory variables were: (1) four habitat types (categorical); (2) pine density within a buffer of 50 m around each nest; (3) land cover (vegetation, ground, rock cover) classified by a random forest model using RGB aerial image 7 cm^2^ resolution; (4) mean vegetation height within a 50-m buffer based on Light Detection and Ranging (LiDAR) [57]; (5) the month that the nests were placed (categorical). Best model was chosen using multi-model inference based on AIC scores.

#### Indirect evidence of predation pressure

We used acoustic monitoring to document mobbing calls of Sardinian warblers and recorded Eurasian Jay calls in the four habitat types as indirect indication for predation events. Preliminary assessment showed that the detection range for Sardinian warbler calls was 50 m from the device. Jays are detected by sound from 200 m during bird count surveys [18], so we assumed they would be readily detected from 50 m. We recorded ambient sounds in 16 plots (four of each habitat type) for five consecutive days from mid-March to May 2015 and 2016. The recordings were made using a mobile recording device (TASCAM model DR-07 MKII; Made in Japan). Because we deployed unused devices of a single model, we expect that the variation among devices is minimal, and did not calibrate our microphones prior to the study. Additionally, our sound data was not used for spatial analysis, which may be biased by differential detection range, but only for species classification, which is based on the signature of sound rather than its magnitude. The recorders were placed in the first 2 h of daylight and recorded at least 6 h of omnidirectional sound. The recording parameters were made at a 16-bit rate and 44.1 kHz. The acoustic analysis was conducted with Raven Pro 1.5 software from the Cornell University Bioacoustics lab [58]. The spectrogram parameters for mobbing calls detection was made in a 592-window size and with 80% overlap in window method Hann. Hann method is a form of smoothing of the sound signal. It raises the cosine curve so that its minima just touch zero. This is done prior to any sound analysis in order to reduce spurious “sidelobes” that appear at frequencies flanking each analysed frequency in a spectrum [59–61].

The Sardinian warbler alarm calls are typically made from repeating tk–tk–tk–tk notes [52]. To detect these mobbing calls automatically we created a detector with the Raven software to detect the single notes of the mobbing calls in the frequency range of 4500 to 6000 Hz duration of 0.01–0.04 s with a minimal time interval of 0.008 s. We used *R* [56] to locate sequences of 20 notes or more (i.e., warbler mobbing sounds) counted all the mobbing calls within a 20-min interval as a single mobbing event. For Eurasian jays we built a detector in frequency range of 1000 to 6000 Hz duration of 0.3–0.5 s with a minimal time interval of 0.5 s. All the automated selections were scanned by hand for any mistakes of the algorithm. In total, we scanned 220 h of recordings. We conducted Kruskal–Wallis test with the *stats* package [56] to find if the number of mobbing events per 2 h was significantly different between the four habitat types.

#### Direct observations

During the two nesting seasons, we conducted direct observations along 10 trails in the first 2 h of the morning in each of the four habitat types. Transects were conducted along fixed 2.5–3 km trails. The transect crossed each vegetation type where artificial nests were placed. Observations were done with a binocular. Transect counts were repeated 8 times throughout each season at 1-week intervals (mid March–May 2015–2016). We recorded Eurasian jay presence and described its activity (e.g., flying, foraging, observing). We used a multivariate abundance model based on a generalized linear model (GLM) to correlate the activity type and habitat type. We assessed goodness of fit using Likelihood Ration Test and Akaike information criterion (AIC) score. We performed a power test analysis to assess the strength of the model. Based on our simulation a minimum of 37 counts are needed to model jay occurrence in relation to observed behaviour and habitat type (simulated degrees of freedom: 26.51; simulated effect size: 0.81 [based on 0.45 of explained variance]; alpha = 0.05; power = 0.95). Analysis was conducted using the *pwr.f2.test* function from ‘pwr’ package available on CRAN [62].

#### Direct evidence of predation with camera traps

We placed 48 camera traps (Bushnell Trophy cam 8MP and 24 Bushnell Trophy cam HD, USA) next to 48 artificial nests with quail eggs for a period of 3 weeks (dates) to directly document predators and predation events behavior. Each camera was stationed next to artificial nests. The cameras were placed 1–1.5 m away from each nest. A previous study showed that the cameras do not bias breeding success [63]. Camera trap photos were examined visually to confirm identification of the marks found on plaster eggs. Photographic data was not analyzed further than that because we did not place cameras at every nest.

## Data Availability

Data collected for this study is stored at and can be requested from Ramat Hanadiv Nature Park.

## Abbreviations

AIC: Akaike information criterion
LiDAR: Light Detection and Ranging

## References

[1] Newbold T, Hudson LN, Hill SLL, Contu S, Lysenko I, Senior RA (2015). Global effects of land use on local terrestrial biodiversity. Nature..

[2] Early R, Bradley BA, Dukes JS, Lawler JJ, Olden JD, Blumenthal DM (2016). Global threats from invasive alien species in the twenty-first century and national response capacities. Nat Commun..

[3] van Kleunen M, Dawson W, Essl F, Pergl J, Winter M, Weber E (2015). Global exchange and accumulation of non-native plants. Nature..

[4] Schirmel J, Bundschuh M, Entling MH, Kowarik I, Buchholz S (2016). Impacts of invasive plants on resident animals across ecosystems, taxa, and feeding types: a global assessment. Glob Chang Biol..

[5] Richardson DM, Rejmánek M (2004). Conifers as invasive aliens: a global survey and predictive framework. Divers Distrib..

[6] Davies KW, Bates JD (2017). Restoring big sagebrush after controlling encroaching western juniper with fire: aspect and subspecies effects. Restor Ecol..

[7] Krannitz PG (2007). Abundance and diversity of shrub-steppe birds in encroachment of ponderosa pine in relation to encroachment of ponderosa pine. Wilson J. Ornithol..

[8] Livingston AC, Varner JM, Jules ES, Kane JM, Arguello LA (2016). Prescribed fire and conifer removal promote positive understorey vegetation responses in oak woodlands. J Appl Ecol..

[9] Bates Jonathan D., Davies Kirk W. (2017). Effects of conifer treatments on soil nutrient availability and plant composition in sagebrush steppe. Forest Ecology and Management.

[10] Bombaci Sara, Pejchar Liba (2016). Consequences of pinyon and juniper woodland reduction for wildlife in North America. Forest Ecology and Management.

[11] Coates Peter S., Prochazka Brian G., Ricca Mark A., Gustafson K. Ben, Ziegler Pilar, Casazza Michael L. (2017). Pinyon and Juniper Encroachment into Sagebrush Ecosystems Impacts Distribution and Survival of Greater Sage-Grouse. Rangeland Ecology & Management.

[12] Richardson D, Rundel P, Price R, Liston A, Strauss S, Millar C, et al. Ecology and biogeography of pinus. Richardson DM, editor. Cambridge: Cambridge University Press; 2000.

[13] Rabinowitch A (1985). Arboreal plant communities of the Mediterranean ecosystems in Israel. Rotem..

[14] Lavi A, Perevolotsky A, Kigel J, Noy-Meir I (2005). Invasion of Pinus halepensis from plantations into adjacent natural habitats. Appl Veg Sci.

[15] Osem Y, Lavi A, Rosenfeld A (2011). Colonization of Pinus halepensis in Mediterranean habitats: consequences of afforestation, grazing and fire. Biol Invasions.

[16] Liphschitz N, Biger G (2001). Past distribution of Aleppo pine (*Pinus halepensis*) in the mountains of Israel (Palestine). Holocene.

[17] Rothschild A. Stopping the forestation in ecological systems in Israel and preserving the natural landscape. SPNI; 2018.

[18] Brosh T, Adam S. Analysis of nesting data at Ramat Hanadiv. Internal Report 2001–2010. 2012.

[19] Shochat E, Abramsky Z, Pinshow B (2002). Breeding bird species diversity in the Negev: effects of scrub fragmentation by planted forests. J Appl Ecol.

[20] Glasser TA, Hadar L (2014). Grazing management aimed at producing landscape mosaics to restore and enhance biodiversity in Mediterranean ecosystems. Options Méditerranéennes Ser A Mediterr Semin..

[21] Schaefer T (2004). Video monitoring of shrub-nests reveals nest predators: Capsule Jays Garrulus glandarius are the most common predators, but carnivorous mammals and some other species also predate nests. Bird Study.

[22] Wegge P, Ingul H, Pollen VO, Halvorsrud E, Sivkov AV, Hjeljord O (2012). Comparing predation on forest grouse nests by avian and mammalian predators in two contrasting boreal forest landscapes by the use of artificial nests. Ornis Fenn..

[23] Fuller RJ, Gregory RD, Gibbons DW, Marchant JH, Wilson JD, Baillie SR (1995). Population declines and range contractions among lowland farmland birds in Britain. Conserv Biol..

[24] Gates JE, Gysel LW (1978). Avian nest dispersion and fledging success in field-forest ecotones. Ecology..

[25] Ricklefs RE (1969). An analysis of nesting mortality in birds. Smithson Contrib Zool..

[26] Yom-Tov Y (1974). The effect of food and predation on breeding density and success, clutch size and laying date of the crow (*Corvus corone* L.). J Anim Ecol..

[27] Martin TE (1988). Habitat and area effects on forest bird assemblages: is nest predation an influence?. Ecology.

[28] Castro-Caro JC, Carpio AJ, Tortosa FS (2014). Herbaceous ground cover reduces nest predation in olive groves. Bird Study.

[29] Howlett JS, Stutchbury BJ (1996). Nest concealment and predation in hooded warblers: experimental removal of nest cover. Auk..

[30] LaManna JA, Hemenway AB, Boccadori V, Martin TE (2015). Bird species turnover is related to changing predation risk along a vegetation gradient. Ecology..

[31] Murcia C (1995). Edge effects in fragmented forests: implications for conservation. Trends Ecol Evol..

[32] Holmes RT, Schultz JC (1988). Food availability for forest birds: effects of prey distribution and abundance on bird foraging. Can J Zool..

[33] Whelan CJ (2001). Foliage structure influences foraging of insectivorous forest birds: an experimental study. Ecology..

[34] Batary P, Baladi A (2004). Evidence of an edge effect on avian nest success. Conserv Biol..

[35] Weidinger K (2009). Nest predators of woodland open-nesting songbirds in central Europe. Ibis (Lond. 1859).

[36] Chalfoun AD, Thompson FR, Ratnaswamy MJ (2002). Nest predators and fragmentation: a review and meta-analysis. Conserv Biol..

[37] Lahti DC (2001). The “edge effect on nest predation” hypothesis after twenty years. Biol Conserv..

[38] McCollin D (1998). Forest edges and habitat selection in birds: a functional approach. Ecography (Cop.)..

[39] Piper Scott D., Catterall Carla P. (2004). Effects of edge type and nest height on predation of artificial nests within subtropical Australian eucalypt forests. Forest Ecology and Management.

[40] Dogra KS, Sood SK, Dobhal PK, Sharma S (2010). Alien plant invasion and their impact on indigenous species diversity at global scale: a review..

[41] Martin Thomas E., Scott Jason, Menge Chris (2000). Nest predation increases with parental activity: separating nest site and parental activity effects. Proceedings of the Royal Society of London. Series B: Biological Sciences.

[42] De Gregorio BA, Weatherhead PJ, Sperry JH (2014). Power lines, roads, and avian nest survival: effects on predator identity and predation intensity. Ecol Evol..

[43] Andren H (1992). Corvid density and nest predation in relation to forest fragmentation: a landscape perspective. Ecology..

[44] Masoero G, Maurino L, Rolando A, Chamberlain D (2016). The effect of treeline proximity on predation pressure: an experiment with artificial nests along elevational gradients in the European Alps. Bird Study..

[45] Welstead K. Factors affecting nest predation artificial and real Sagebrush Brewer’s Sparrow (*Spizella breweri breweri*). British Columbia; 2002.

[46] Schmidt KA (1999). Foraging theory as a conceptual framework for studying nest predation. Oikos.

[47] Browning DM, Archer SR, Asner GP, McClaran MP, Wessman CA (2008). Woody plants in grasslands: post-encroachment stand dynamics. Ecol Appl.

[48] Hadar L, Perevolotsky A (1989). The native flora in Ramat-Hanadiv. Ramat Hanadiv as a parable.

[49] Emery NJ, Clayton NS (2004). The mentality of crows: convergent evolution of intelligence in corvids and apes. Science.

[50] Shaw RC, Plotnik JM, Clayton NS (2013). Exclusion in corvids: the performance of food-caching Eurasian jays (*Garrulus glandarius*). J Comp Psychol..

[51] Paul Shaw IN (2009). Ancient egyptian materials and technology.

[52] Shirihai H, Gargallo G, Helbig AJ, Harris A, Kirwan GM, Svensson L (2001). Sylvia warblers.

[53] Elbroch M, Marks E. In: Eleanor M, Boretos CD. Bird tracks & sign : a guide to North American species. Mechanicsburg: Stackpole Books; 2001.

[54] Wang Y, Naumann U, Wright S, Warton D, Wang MY, Rcpp I, et al. Package ‘mvabund.’ Cran R. 2018.

[55] Wang Y, Naumann U, Wright ST, Warton DI (2012). mvabund—an R package for model-based analysis of multivariate abundance data. Methods Ecol Evol..

[56] R Core Team (2015). A language and environment for statistical computing.

[57] Bar Massada A, Kent R, Blank L (2012). Automated segmentation of vegetation structure units in a Mediterranean landscape. Int J Remote Sens..

[58] Bioacoustics Research Program. Raven Pro: Interactive Sound Analysis Software (Version 1.4) [Computer software]. Ithaca, NY: The Cornell Lab of Ornithology; 2011. Available from http://www.birds.cornell.edu/raven.

[59] Berryman Fiona, Pynsent Paul, Cubillo James (2004). The effect of windowing in Fourier transform profilometry applied to noisy images. Optics and Lasers in Engineering.

[60] Podder P, Zaman Khan T, Haque Khan M, Muktadir Rahman M (2014). comparative performance analysis of Hamming, Hanning and Blackman window. Int J Comput Appl.

[61] Wickramarachi P (2003). Effects of windowing on the spectral content of a signal. Sound Vib..

[62] Champely S, Ekstrom C, Dalgaard P, Gill J, Weibelzahl S, Anandkumar A, et al. Package “pwr” (1.2–2). 2018;1–22. https://cran.r-project.org/web/packages/pwr/pwr.pdf.

[63] Herranz J, Yanes M, Suárez F (2002). Does photo-monitoring affect nest predation?. J Field Ornithol..

